# Design and Microfabrication of An On-Chip Oocyte Maturation System for Reduction of Apoptosis

**DOI:** 10.22074/cellj.2021.7056

**Published:** 2021-03-01

**Authors:** Behnaz Sadeghzadeh Oskouei, Siavash Zargari, Parviz Shahabi, Marefat Ghaffari Novin, Maryam Pashaiasl

**Affiliations:** 1. Department of Midwifery, School of Nursing and Midwifery, Tabriz University of Medical Sciences, Tabriz, Iran; 2. Department of Electrical and Computer Engineering, University of Tabriz, Tabriz, Iran; 3. Department of Physiology, School of Medicine, Tabriz University of Medical Sciences, Tabriz, Iran; 4. Cellular and Molecular Biology Research Center, School of Medicine, Shahid Beheshti University of Medical Sciences, Tehran, Iran; 5. Drug Applied Research Center, Tabriz University of Medical Sciences, Tabriz, Iran

**Keywords:** Assisted Reproductive Technology, Apoptosis, *In vitro* maturation, Microfluidics, Oocyte

## Abstract

**Objective:**

In customary assisted reproductive technology (ART), oocyte culture occurs in static micro drops of Petri dishes with vast media volume; while, the *in vivo* condition is dynamic. In this study, we aimed to improve the maturation efficiency of mammalian oocytes by designing an optimal microchamber array to obtain the integration of oocyte trapping and maturation within a microfluidic device and evaluate the role of microfluidic culture condition in lipid peroxidation level of the culture medium, *in vitro* matured oocytes apoptosis, and its comparison with the conventional static system.

**Materials and Methods:**

In this experimental research, immature oocytes were collected from ovaries of the Naval Medical Research Institute (NMRI) mice. Oocytes were randomly laid in static and dynamic (passive < active)*in vitro* maturation culture medium for 24 hours. The lipid peroxidation level in oocyte culture media was assessed by measuring the concentration of malondialdehyde (MDA), and the rate of apoptosis in *in vitro* matured oocytes was assessed by the TUNEL assay after a-24 hour maturation period.

**Results:**

The MDA concentration in both dynamic oocyte maturation media were significantly lower than the static medium (0.003 and 0.002 vs. 0.13 µmol/L, P<0.01). Moreover, the rate of apoptosis in matured oocytes after a-24 hour maturation period was significantly lower in passive dynamic and active dynamic groups compared with the static group (16%, 15% vs. 35%, P<0.01).

**Conclusion:**

The dynamic culture for *in vitro* oocyte maturation (IVM) improves the viability of IVM oocytes in comparison with the static culture condition.

## Introduction

*In vitro* maturation (IVM) of mammalian oocytes is an important infertility treatment with substantial assurance. At present, IVM methods are very effective when used for mice and still regarded experimental in the human clinic due to suboptimal fertilization rates and embryo quality. Therefore, in order to achieve human embryos that have the same developmental potential as embryos resulting from standard IVF, assisted reproductive technology (ART) must improve increasingly in the field of IVM (1). Nevertheless, ART is quite expensive, and it has a generally low success rate (2). One of the considerable differences between *in vivo* and *in vitro* conditions for the oocyte/embryo is oxygen tension. Accordingly, *in vitro* culture is kept up with higher concentrations of O_2_ in comparison with the *in vivo* culture, and therefore, it can result in the increased production of reactive oxygen species [ROS, (3)]. The relatively high oxygen concentrations in the *in vitro* microenvironment of the cells may disturb the equilibrium between the formation of reactive oxygen species and antioxidants, causing a stressful condition called to oxidative stress (1).

Oxidative stress is involved in a wide range of biological events, including oxidation of amino acids and nucleic acids, apoptosis, necrosis, and membrane lipids peroxidation. The plasma membrane of the mammalian oocytes is a rich source of unsaturated fatty acids and vulnerable to ROS-related lipid peroxidation. The cellular structure and metabolic functions of the oocyte can be reduced as a result of lipid peroxidation, which is caused by ROS. The level of lipid peroxidation is defined by measuring the level of malondialdehyde (MDA), which is a stable lipid peroxidation product (4).

Cell death in the form of apoptosis is a physiologic phenomenon, occurring during several processes, including gametogenesis/embryogenesis. Although apoptosis takes place as an ordinary component of *in vivo* development; however, it is more likely to occur during suboptimal *in vitro* culture conditions (1).

To date, all efforts and advances made in this area caused modifications in the ingredients of culture media, while there is no significant change in the physical instruments utilized for handling and manipulation of oocytes/embryos in ART research centers (5, 6). 

Unfortunately, embryology laboratories still use petri dishes and fine-bore-glass-pipettes as static culture systems (2). Such conditions result in the induction of numerous alterations in oocytes and embryos. There are more controllable factors, such as media components, protein supplements, and embryo density. However, all of these compounds are static for several days and cells have to reside for a long time; therefore, cellular damages, such as epigenetic alterations may occur. It seems that simulating *in vivo* dynamic conditions in the culture medium increases its culture quality (7). Obviously, the dynamic environment of tubal/uterine produces a unique condition capable of supporting embryo development and it can also regulate gene expression (8) and interrupt cell-surface gradients on embryos (9). These gradients, such as potassium, calcium, and oxygen also exist in conventional static culture conditions, possibly through the secretion of trophic autocrine/paracrine factors; however, because of the nature of static conditions, the previously mentioned gradients cannot be disturbed, and therefore would not provide a more homogenous environment similar to *in vivo* dynamic culture systems [DCSs, (3)]. It is assumed that IVM of oocytes in a microfluidic environment can resemble *in vivo* conditions for oocyte development more closely and thus benefiting the maturation of efficient oocyte and subsequent embryo development. It is worth mentioning that each individual event during oocyte maturation can highly influence the subsequent embryonic development (10, 11).

Accordingly, the main goal of this study is to evaluate the effect of microfluidic culture condition on MDA concentration in culture media, *in vitro* matured oocytes apoptosis, and its correlation with conventional static system. A microfluidic device made (Patent No. 96301) by colleagues were used as dynamic culture condition (12). The success and applicability of the present device in the reduction of apoptosis and MDA production are the focus of this study.

## Materials and Methods

### Study design and animals

In this experimental study, immature oocytes were obtained from ovaries of the Naval Medical Research Institute (NMRI) mice (Pasteur Institute, Tehran, Iran) with an age range of 6-8 weeks, and sperm samples were acquired from male mice with an age range of 8-10 weeks. They were kept under a controlled condition with 12- hour light/dark cycle, constant temperature, and relative humidity conditions with free access to water and food. All experiments procedures were carried out according to the Ethics Guidelines of Tabriz University of Medical Sciences (Registration number: 5/79/462). Oocytes were randomly laid in static and dynamic (passive and active) *in vitro* maturation culture media for 24 hours. The lipid peroxidation level in oocyte culture media was assessed by measuring the MDA level, and the rate of apoptosis in the *in vitro* matured oocytes was assessed by the TUNEL assay after a-24 hour maturation period.

### Computational model

COMSOL multiphysics software was employed for modeling and simulation of IVM microfluidic device to optimize the design, improve the performance, and reduce the process time. COMSOL is an interactive environment for modeling and simulating scientific and engineering problems. It permits conventional physics-based user interfaces and coupled systems of partial differential equations (PDEs) and enables the simulation of designs and devices dependent on electromagnetics, structural mechanics, acoustics, fluid flow, heat transfer, and chemical engineering behavior.

Herein, we built finite element models and used 2D and 3D simulations to study the flow behavior in microchannel and microchambers. The model is based on the steadystate Navier–Stokes equation for incompressible Newtonian fluid (13):

(1) $$\frac{\partial \rho }{\mathrm{\partial t}}+\mathrm{\nabla .(\rho u)}=0$$

(2) $$\rho \frac{\mathrm{\partial u}}{\mathrm{\partial t}}+\mathrm{\rho (u.\nabla )u}+\mathrm{\nabla p}-{\mu \nabla }^{2}u-(\lambda +\mu )\nabla (\mathrm{\nabla .u})=f$$

Where u is the velocity vector field, p the pressure, ρ the medium density, μ the dynamic viscosity, λ the molecular mean free path, and f the body force. 

In addition, the computational fluid dynamics was utilized to anticipate the wall shear stress as a function of the channel width and flow rate. Since the channel length was significantly large compared with the channel height, the system could be effectively modeled using a 2D simulation. Using these conditions, the maximum shear stress applied to the cell (τ_s_ ) could also be assessed by this equation (14):

(3) $$\tau_{s}=\frac{\left(6\times 2.95\mathrm{\mu Q}\right)}{H^{2}}$$

Where H is channel height, the medium viscosity, and Q the flow rate.

### Device design and fabrication

The device was optimally designed using a finite element method (FEM) under the defined criteria and then fabricated using standard soft lithography procedures in our previously published work (15).

Master molds patterned with 200-µm thick resist were made by patterning a negative photoresist (SU-8 2050; Microchem, MA, USA) on a standard PCB substrate. The main biochip is then injection molded into the associated masters by pouring uncured PDMS pre-polymer (Sylgard 184; Dow Corning, MI, USA) solution over the masters. PDMS was chosen for this microfluidics system microfabrication because of its favorable mechanical properties, optical transparency, biocompatibility (16, 17), and straightforward manufacturing by rapid prototyping (12).

To remove microbubbles, the solution was then degassed in a desiccator using a single stage vacuum pump and kept in the oven at 70˚C for 90 minutes, and then peeled off from the master. To improve the adhesion, the upper and lower PDMS layers were surface-treated utilizing a corona discharge gun for 4 minutes, and then bonded under the pressure. The bonded layers were then heated in the oven up to 70˚C for 20 minutes to reinforce the bonding. Two sampler tips were then fixed on the upper layer as the inlet and outlet reservoirs.

**Fig.1:**
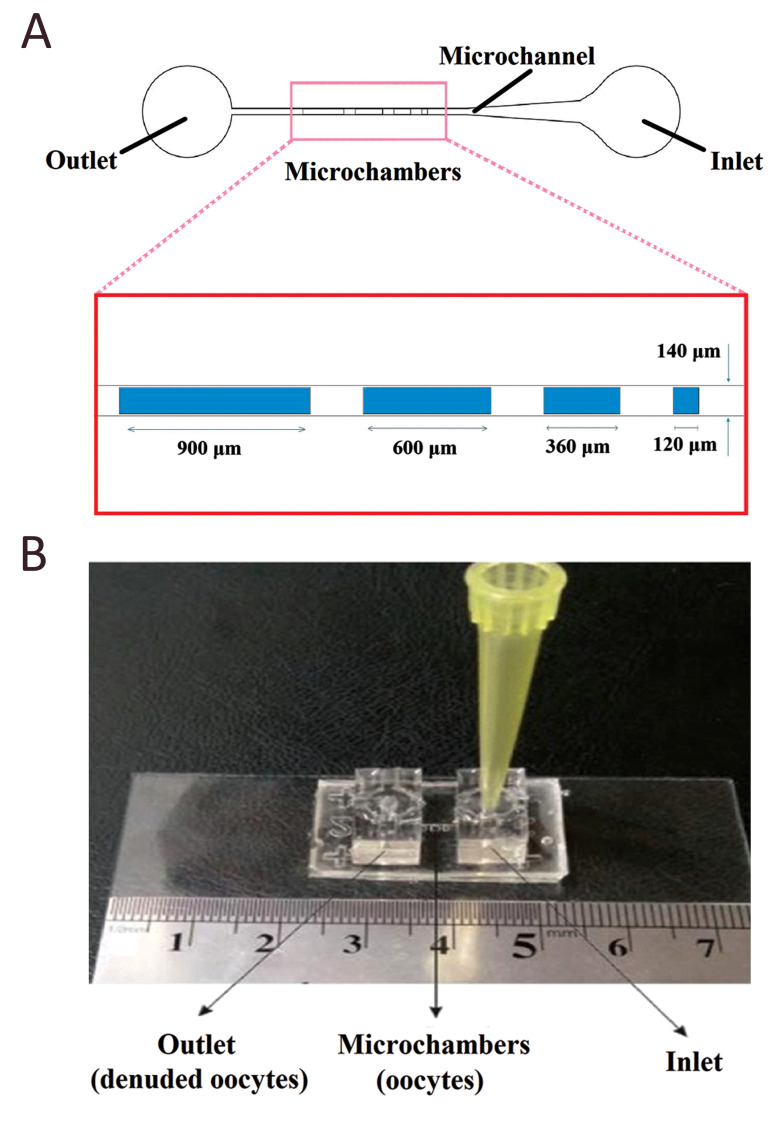
Schematic view of the proposed microfluidic device. **A.** Illustration of the microfluidic device and microchamber array (15), and **B.** Fabicated microfluidic device (12).

Two types of devices (Passive and Active) consist of two PDMS layers, with a microchannel in the upper layer and a microchamber array in the lower one. The microchannel had a 7-mm length, 140-μm width, and 200-μm depth to transport oocytes, and the inlet and outlet were connected to the microchannel (Fig.1A). Square-shaped microchambers had a 120-μm width, 120-μm, 360-μm, 600-μm and 900-μm length, respectively, at a 250-μm interval distance to trap oocytes. The four microchambers were designed to compare their performance in oocyte capture and developmental competency of cumulus oocyte complexes (COCs) in groups of 1, 3, 5, and 7. 

During the experiment, oocytes were loaded into the funnel shape inlet port, transported through the microchannel and lodged in the microchamber array. The design of the present culture microfunnels were selected rather than culture channels to minimize excessive fluid mechanical stress that may be associated with passage through narrow channels in previous studies (18). The illustration of the microfluidic device and microchamber array and the final assembled device are shown in Figure 1A, B.

### Superovulation, oocyte collection, and *in vitro* maturation

To obtain immature oocyte, female mice were induced by superovulation. They were injected intraperitoneally (i.p.) with 10 IU of PMSG (Gonaser®, Laboratorios Girona, Spain). Oocytes were retrieved 46–48 hours after PMSG injection. 

Animals were sacrificed by cervical dislocation and their ovaries were removed and placed in tissue culture dishes (BD Falcon, 35 x 10 mm) containing human tubal fluidHEPES buffered (GC-HTF W/HEPES; Genocell ideal, Iran) supplemented with 10% (v/v) qualified fetal bovine serum (FBS, Gibco, Invitrogen, South America). COCs were released by follicular puncturing with the aid of a pair of 28G needles under a stereomicroscope (Olympus, Japan) (19). Only cumulus intact oocytes in germinal vesicle (GV) stage evenly granulated cytoplasm were selected and moved to maturation medium (20). After several washes, just fully expanded COCs (19, 20) were divided into dynamic (passive and active) and static culture system groups, randomly and synchronously.

In dynamic groups, COCs were loaded into microfluidic chips pre-filled with 15-20 µL of human tubal factor (HTF) medium supplemented with 10% (v/v) qualified fetal bovine serum, 10 µg/mL follicle stimulating hormone (FSH), 10 µg/mL luteinizing hormone (LH), and 1 µg/mL estradiol17ß (Sigma Chemical company, St. Louis, MO, USA) as an IVM medium (5 oocytes/15 µL medium) and left to rest for 5 minutes. COCs were incubated in a humidified atmosphere with 5% CO_2_ at 37˚C for 20-24 hours (21).

**Fig.2:**
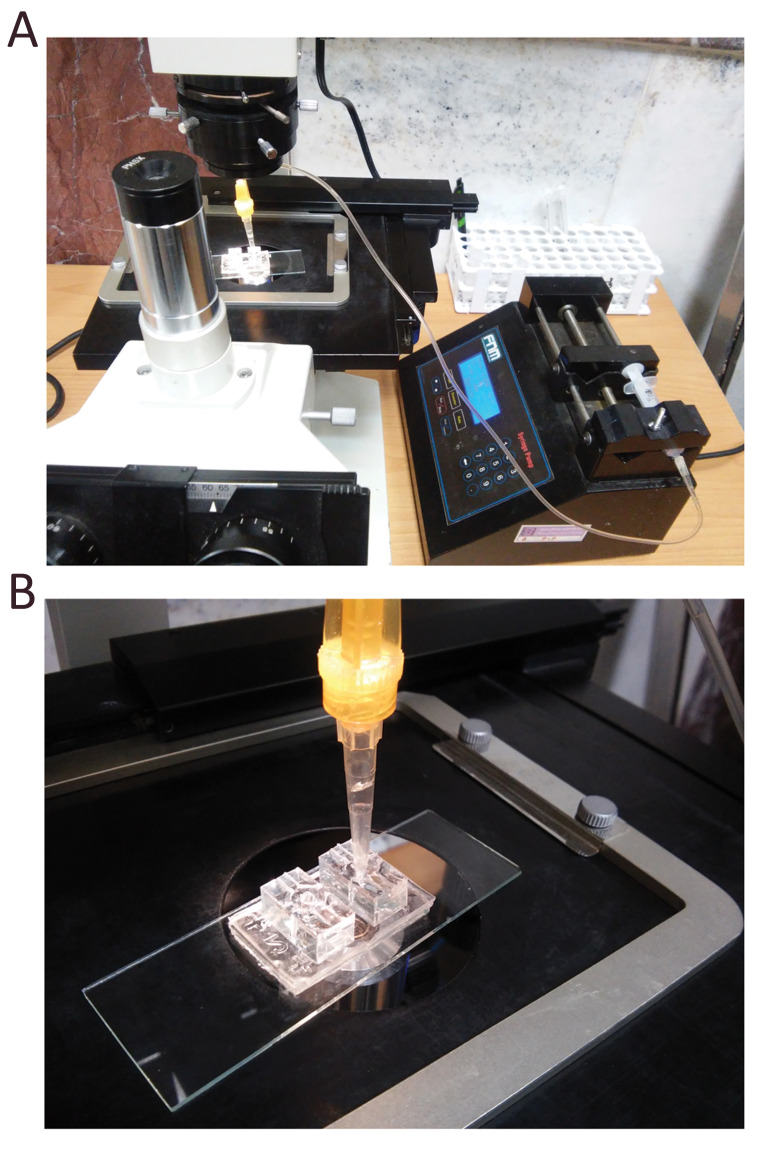
Schematic view of the experimental perfusion system comprising. **A. **a syringe pump and **B.** the fabricated microfluidic chip.

As shown in Figure 2A, a syringe pump was connected to the inlet reservoir to generate a negative-pressure driven flow. The flow lasted for 2 minutes at an inlet velocity of 0.5 mm/s to guide oocytes into the microchamber array area.

In the active dynamic group, we utilized a low flow rate peristaltic pump (Langer Instruments, USA), which produced pulsatile fluid movement (1µL/h), but in the passive dynamic group, we did not use any pump, and fluid movement was made by gravity

In the static group, COCs were set in several droplets of the IVM medium (15 oocytes/50 µL medium under mineral oil) (20, 22) in tissue culture dishes and after that incubated in a similar condition used for experimental (dynamic) groups for 24 hours. Then, apoptosis in two groups was assessed under a fluorescent microscope (Labomed, USA).

### TUNEL assay

The TUNEL assay and propidium iodide staining were performed to evaluate DNA fragmentation in oocytes. Following several washings in PBS (Gibco, Grand Island, NY, USA), oocytes were fixed in 3.7% paraformaldehyde solution (Wako, Japan), treated with 0.1% Triton X-100 solution (Sigma, Germany) for 40 minutes and exposed to the blocking solution at 4˚C overnight. Then, oocytes were primarily incubated in TUNEL solution (Roche, Germany) at 37˚C for an hour according to the manufacturer’s guidelines. Negative control oocytes were incubated only in the fluorescent solution lacking the enzyme to ensure the absence of labeling. For the positive control, a number of oocytes prior to the incubation with TUNEL staining solution were incubated with 50 µg/ml of the DNase I solution (Sigma, Germany) for one hour and then treated with the TUNEL solution. Oocytes were stained with 50 µg/ml of the propidium iodide (Sigma, Germany) solution for 20 minutes to label nuclei and examined under a fluorescent microscope (Labomed, USA).

### TUNEL assay

The TUNEL assay and propidium iodide staining were performed to evaluate DNA fragmentation in oocytes. Following several washings in PBS (Gibco, Grand Island, NY, USA), oocytes were fixed in 3.7% paraformaldehyde solution (Wako, Japan), treated with 0.1% Triton X-100 solution (Sigma, Germany) for 40 minutes and exposed to the blocking solution at 4˚C overnight. Then, oocytes were primarily incubated in TUNEL solution (Roche, Germany) at 37˚C for an hour according to the manufacturer's guidelines. Negative control oocytes were incubated only in the fluorescent solution lacking the enzyme to ensure the absence of labeling. For the positive control, a number of oocytes prior to the incubation with TUNEL staining solution were incubated with 50 µg/ml of the DNase I solution (Sigma, Germany) for one hour and then treated with the TUNEL solution. Oocytes were stained with 50 µg/ml of the propidium iodide (Sigma, Germany) solution for 20 minutes to label nuclei and examined under a fluorescent microscope (Labomed, USA).

### MDA assay

In order to determine malondialdehyde (MDA) concentration in oocyte culture media, we used the MDA assay method. Lipid peroxidation was measured by the reaction of thiobarbituric acid (TBA) with MDA. The content of MDA was determined spectrofluorometrically using a spectrofluorometer (PG instruments T70 UV/VIS, 532 nm).

The MDA fluorescence intensity of oocyte was evaluted utilizing different concentrations of tetraethoxy-propane as the standard. The results are expresed as µmol MDA/L of the culture medium.

### Statistical Analysis

All statistical analyses were implemented utilizing Service Provisioning System Software (SPSS) 22 for windows (SPSS, Chicago, IL, USA). The *in vitro* maturation and apoptosis rate in static and dynamic groups were compared by one-way analysis of variance (ANOVA) followed by Tukey, s Post Hoc test. Data are expressed as means ± SE. Differences at P-value of less than 0.05 were considered statistically significant.

## Results

### Computational analysis of the shear stress profile

We used computational fluid dynamics (CFD) features of COMSOL multiphysics to predict velocity profiles and shear stress patterns in our proposed structure. The culture medium (HTF) was modeled at a density of 1000 kg/m3 and a dynamic viscosity of 0.001 Pa.s (23). A uniform inlet velocity of 0.5 mm/s was used to simulate the experimental conditions. A zero pressure condition was applied to the outlet. No-slip boundary conditions were applied in the microchannel walls. Steadystate 2D velocity profiles and streamlines were obtained.

**Fig.3:**
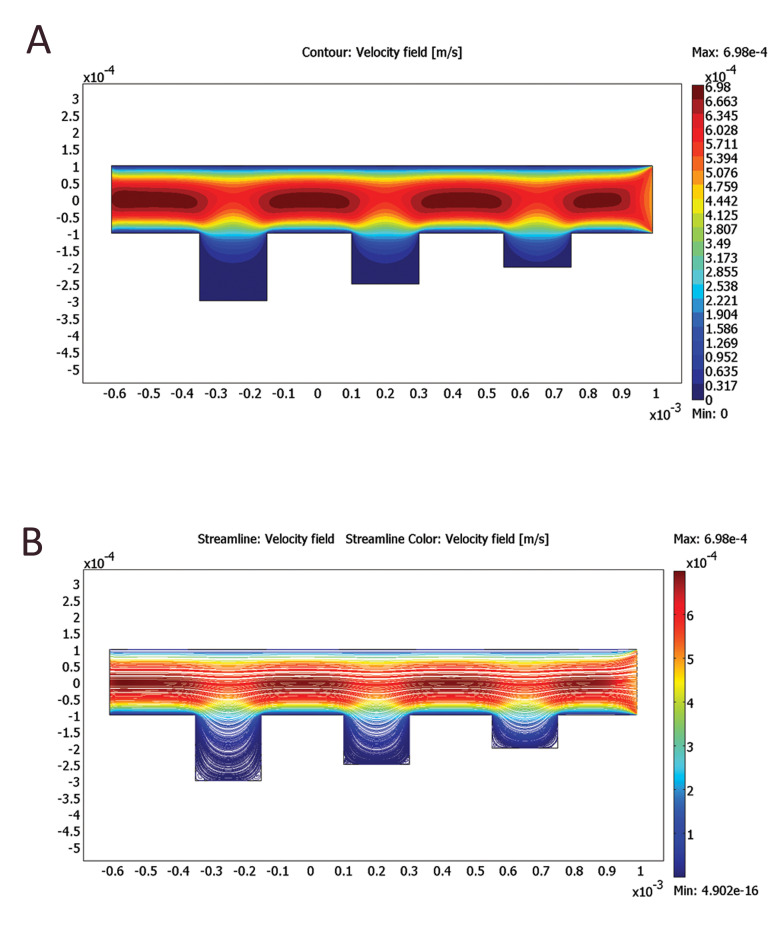
Simulation results obtained from COMSOL Multiphysics software. **A. **Velocity contours for microchambers with different lengths, and **B. **Streamlines for microchambers with different lengths.

Figure 3 represent velocity contours and streamlines of the laminar flow, resulting from the 2D simulation under an average inlet velocity of 0.5 mm/s. As shown in Figures, higher flow penetration in the chambers was achieved in larger chambers (600 and 900 μm in length) compared with the smaller ones (120 and 360 μm in length). This was supposed to have significant impacts on oocyte maturation due to better media circulation along the chambers.

Another vital parameter is the total stress parameters applied to the oocyte during maturation period. Extra tangential forces have degrading effects on the oocytes and could even cause degeneration (24). These forces could be measured in terms of the shear stresses from mechanical point of view. The resulting shear stress parameters within the microchambers are also calculated in the COMSOL using equation (3).

**Fig.4:**
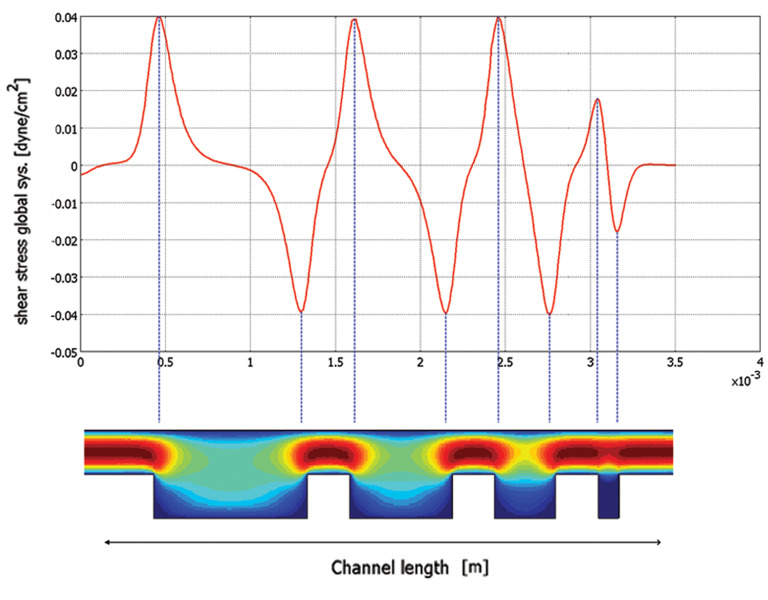
The shear stress profile in the microchannel.

Figure 4 represents the shear stress profile for the different microchambers lengths. Here the extreme values (positive and negative peaks) correspond to chamber walls where an absolute maximum shear stress occurs over the oocytes and the sign of shear stress only explain changes in the direction of flow. For an inlet velocity of 0.5 mm/s, the maximum wall shear stress inside the microchannel was 0.04 dyne/cm2, which was significantly lower than the average wall shear stress amplitude that an oocyte can tolerate (1.2 dyne/cm2) (24). According to the simulation results (Fig.3A, B), it can be deduced that the fluid velocity inside the microchambers is much lower than the fluid velocity inside the main channel (red and blue colors indicate the highest and lowest velocities, respectively). As a result, when oocytes are placed inside the microchambers, very small shear stress is applied, and the maximum amount of shear stress is applied to the oocytes only at the beginning and end of each microchamber.

### Qualitative observations

**Fig.5:**
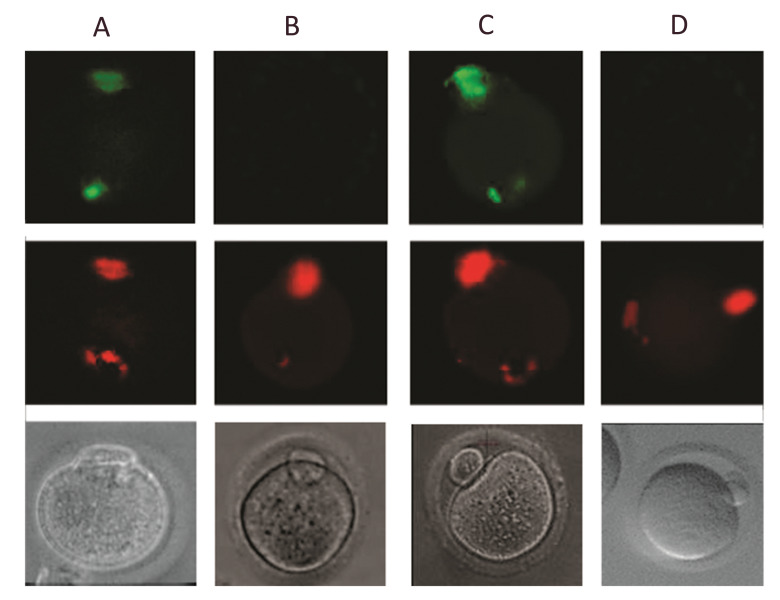
Microscopic view of mouse stage metaphase II oocytes under a fluorescent and stereo microscope. **A.** Cell nuclei were stained as greencolored after incubation with DNase I (positive control).** B.** Microscopic view of mouse oocytes under a fluorescent microscope; no cells were marked in the absence of terminal deoxy transferase but cells with stained nuclei with the propidium iodide (negative control). **C.** Microscopic view of an apoptotic cell.** D.** Microscopic view of a non-apoptotic cell. Upper figures were incubated with DNase I; middle figures were incubated with propidium iodide, and the below figures were not incubated.

Figure 5 indicates that all positive control oocytes were labeled by TUNEL assay solution and appeared as greencolored. None of the negative control oocytes were labeled by TUNEL assay solution and only counterstained by propidium iodide and observed as red-colored (Fig.5B). As shown in Figure 5C, *in vitro* matured oocytes were stained to assess apoptosis, i.e., the apoptotic cells were well labeled with TUNEL staining solution and quite distinct from the non-apoptotic cells (Fig.5D). 

### The apoptosis rate in matured oocytes after a 24-hour maturation period

**Fig.6:**
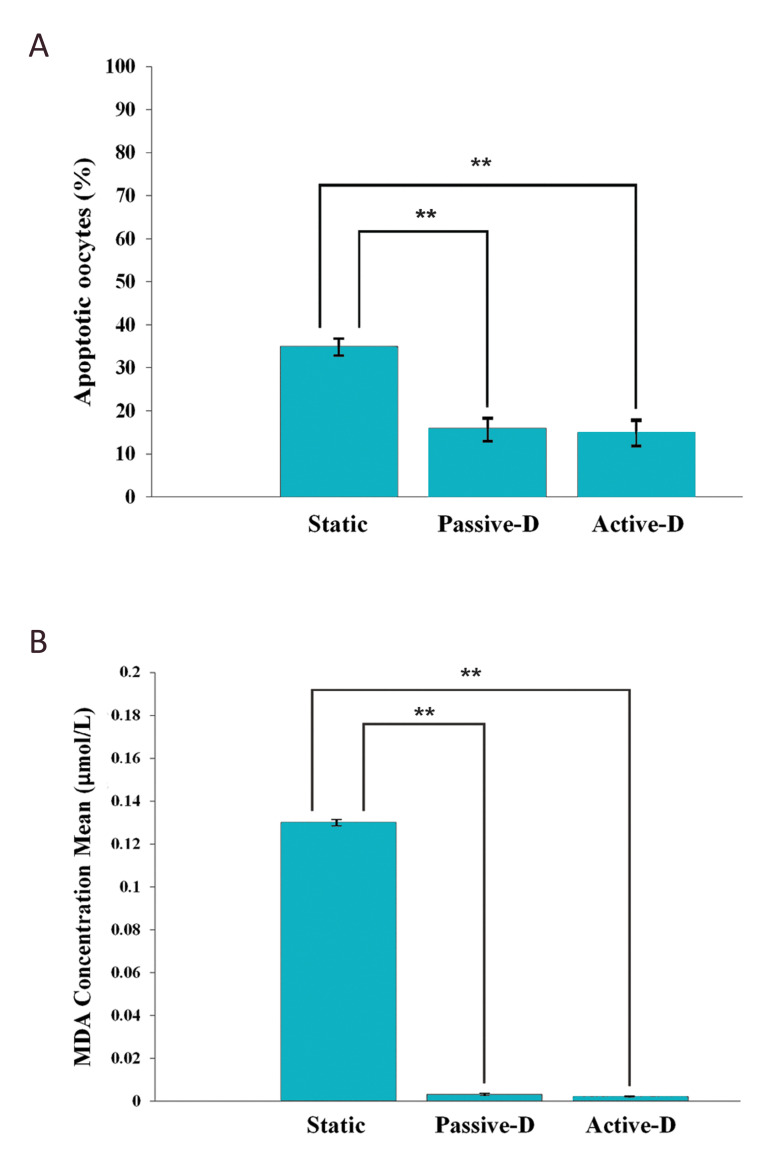
Graphical representation of the rate of apoptosis and MDA assay outcomes. **A.** The number of apoptotic oocytes in three groups. **B.** The concentration of MDA of three groups in oocyte culture media. Data are expressed as mean ± SE. Asterisks indicate statistically significant differences relative to the static group. MDA; malondialdehyde, and **; (P<0.01).

As shown in Figure 6A, the rate of apoptosis in matured oocytes after a 24-hour maturation period showed a significant difference in passive dynamic and active dynamic groups compared with the static group (16%, 15% vs. 35%, P<0.01).

### MDA assay

The means ± SD of lipid peroxidation values, as determined by the MDA assay, in cultured oocytes are shown in Figure 6B.

## Discussion

This paper describes the effect of dynamic culture system on immature oocyte development by microfluidic chip, exclusively designed and constructed by the present authors (Patent No. 96301) (12). For having a good quality embryo, high quality matured oocyte is necessary. It has been reported that a dynamic culture of preimplanted embryos plays a role in successful implantation and ongoing normal pregnancies due to obtaining the developmental competence. However, few studies focused on the dynamic culture of immature oocytes (25). To do ART procedures, such as intracytoplasmic sperm injection (ICSI), the induction ovulation was performed. Frequently, a number of oocytes obtained after stripping contained at least one immature oocyte (i.e., oocytes at the metaphase I (MI) or GV stage of development) (26). Therefore, the rescue of immature oocytes would be critical. Conventional culture condition is static; however, the *in vivo* condition is dynamic. The dynamic condition is optimal for cell development (12). The present findings showed that the dynamic culture during IVM efficiently improved oocyte development. Embryos acquired from dynamic matured oocytes demonstrated higher development compared with the blastocyst stage, which is consistent with previous studies (27, 28).

Oocyte maturation is the most significant stage in ART protocols, since it determines subsequent successful fertilization, zygote formation, and suitable transition to the blastocyst stage, as well as appropriate implantation (8, 29).

For achieving a high quality embryo, efficient matured oocyte would be necessary. The application of the lab-ona-chip (LOC) system in reproductive biology provides new possibilities for the development of techniques to assess the developmental competency of mammalian oocytes. This system may provide controllable microenvironments specialized for embryo development in addition to an automated platform for performing the multiple IVF steps (30-33).

Willadsen first reported on the importance of the microenvironment and embryo handling/culture in the 1970s (33). Choi developed a microfluidic device capable of selecting normal oocytes with relatively high specificity (34). Similarly, intrinsic sperm mobility and microfluidic laminar flow were used to isolate motile spermatozoa from non-motile sperm, debris and seminal plasma (35, 36). Zeringue developed a microfluidic platform for the control of embryo positioning, movement, and zona pellucida removal for chimeric and transgenic production (37). Although these devices provide convenient handling properties for spermatozoa, oocytes, and embryos, they did not address the potential of microfluidics technology in their developmental competency. Therefore, the present research has been planned to investigate the oocyte maturation improvement. 

Our results from this comparative controlled research proposed that the microenvironment obtained by microfluidics supports enhanced the immature oocyte development compared with the conventional static culture conditions and decreased apoptosis rate and MDA level in the dynamic condition in comparison with the static one. The greatest development of immature oocytes has been shown to occur in small volumes or in the presence of multiple similar cells, which is likely due to the beneficial effects of autocrine oocyte secreted factors (OSFs) (38, 39). Oocyte culture in dynamic condition, due to small microenvironment, result in better effect of autocrine factors (12).

Our findings have also affirmed those of previous studies. We demonstrated that fluid movement and mechanical agitation of immature oocytes during dynamic culture could improve their development. We observed significantly lower apoptosis rate in dynamic culture groups compared to the static group (P<0.01). 

Moreover, the MDA level in dynamic groups was considerably low in comparison with the static group (P<0.01). Previous studies did not provide strong evidence concerning oxidative stress in oocytes. The relatively high oxygen concentrations in the preimplantation embryos disturb the balance between the formation of reactive oxygen species and antioxidants, leading to oxidative stress (40). Esfandiari et al. showed a significant correlation (P<0.0001) between the level of ROS in different embryo culture media and they reported that a positive association between the levels of ROS at 24 hours and the blastocyst apoptosis rate (1). To date, no study compared the level of MDA and apoptosis rate in two culture media (static and dynamic) by the national production, but our results suggest such a survey would be warranted. 

## Conclusion

In this study, we utilized a microfluidic device for MDA assay and apoptosis rate of *in vitro* matured oocytes compared with the static system. Our results show that the utilization of microfluidic device in order to provide a dynamic culture condition has optimal effects on apoptosis and the decrease of MDA production. In present study, immature oocytes rescued in dynamic condition.

## References

[1] Esfandiari N, Falcone T, Agarwal A, Attaran M, Nelson DR, Sharma RK (2005). Protein supplementation and the incidence of apoptosis and oxidative stress in mouse embryos. Obstet Gynecol.

[2] Beebe DJ, Glasgow IK, Wheeler MB (2001). Microfluidic embryo and/or oocyte handling device and method.Google Patents.

[3] Agarwal A, Gupta S, Sikka S (2006). The role of free radicals and antioxidants in reproduction. Curr Opin Obstet Gynecol.

[4] Agarwal A, Saleh RA, Bedaiwy MA (2003). Role of reactive oxygen species in the pathophysiology of human reproduction. Fertil Steril.

[5] Vajta G, Rienzi L, Cobo A, Yovich J (2010). Embryo culture: can we perform better than nature?. Reprod Biomed Online.

[6] Smith GD, Takayama S, Swain JE (2012). Rethinking in vitro embryo culture: new developments in culture platforms and potential to improve assisted reproductive technologies. Biol Reprod.

[7] Gardner DK, Kelley RL (2017). Impact of the IVF laboratory environment on human preimplantation embryo phenotype. J Dev Orig Health Dis.

[8] da Rocha AM, Smith GD (2012). Culture systems: fluid dynamic embryo culture systems (microfluidics). Methods Mol Biol.

[9] Matsuura K, Naruse K (2012). Use of silicone elastomer-based microfluidic devices and systems in reproductive technologies.Advanced Elastomers-Technology, Properties and Applications: InTech.

[10] Hendriksen PJ, Vos PL, Steenweg WN, Bevers MM, Dieleman SJ (2000). Bovine follicular development and its effect on the in vitro competence of oocytes. Theriogenology.

[11] Spindler RE, Pukazhenthi BS, Wildt DE (2000). Oocyte metabolism predicts the development of cat embryos to blastocyst in vitro. Mol Reprod Dev.

[12] Sadeghzadeh Oskouei B, Pashaiasl M, Heidari MH, Salehi M, Veladi H, Ghaderi Pakdel F (2016). Evaluation of mouse oocyte in vitro maturation developmental competency in dynamic culture systems by design and construction of a lab on a chip device and its comparison with conventional culture system. Cell J.

[13] Pelesko JA, Bernstein DH (2002). Modeling mems and nems.Boca Raton, Florida: CRC press.

[14] Gaver DP 3rd, Kute SM (1998). A theoretical model study of the influence of fluid stresses on a cell adhering to a microchannel wall. Biophys J.

[15] Zargari S, Veladi H, Sadeghzadeh B, Shahabi P, Frounchi J (2016). A Microfluidic Chip for In Vitro Oocyte Maturation. Sensor Lett.

[16] Quake SR, Scherer A (2000). From micro-to nanofabrication with soft materials. Science.

[17] Johnson TJ, Ross D, Gaitan M, Locascio LE (2001). Laser modification of preformed polymer microchannels: application to reduce band broadening around turns subject to electrokinetic flow. Anal Chem.

[18] Heo YS, Cabrera LM, Bormann CL, Shah CT, Takayama S, Smith GD (2010). Dynamic microfunnel culture enhances mouse embryo development and pregnancy rates. Hum Reprod.

[19] Kawamura K, Kawamura N, Mulders SM, Sollewijn Gelpke MD, Hsueh AJ (2005). Ovarian brain-derived neurotrophic factor (BDNF) promotes the development of oocytes into preimplantation embryos. Proc Natl Acad Sci USA.

[20] Salimi M, Salehi M, Masteri Farahani R, Dehghani M, Abadi M, Novin MG (2014). The effect of melatonin on maturation, glutathione level and expression of H MGB1 gene in brilliant cresyl blue (BCB) stained immature oocyte. Cell J.

[21] Eddings MA, Johnson MA, Gale BK (2008). Determining the optimal PDMS-PDMS bonding technique for microfluidic devices. Journal of Micromechanics and Microengineering.

[22] Martin-Coello J, Gonzalez R, Crespo C, Gomendio M, Roldan ER (2008). Superovulation and in vitro oocyte maturation in three species of mice (Mus musculus, Mus spretus and Mus spicilegus). Theriogenology.

[23] Singh H, Ang ES, Lim T, Hutmacher DW (2007). Flow modeling in a novel non-perfusion conical bioreactor. Biotechnol Bioeng.

[24] Xie Y, Wang F, Zhong W, Puscheck E, Shen H, Rappolee DA (2006). Shear stress induces preimplantation embryo death that is delayed by the zona pellucida and associated with stress-activated protein kinase-mediated apoptosis. Biol Reprod.

[25] Fauci LJ, Dillon R (2006). Biofluidmechanics of reproduction. Annu Rev Fluid Mech.

[26] Johnson JE, Higdon HL 3rd, Boone WR (2008). Effect of human granulosa cell co-culture using standard culture media on the maturation and fertilization potential of immature human oocytes. Fertil Steril.

[27] Smith GD, Takayama S (2007). Gamete and embryo isolation and culture with microfluidics. Theriogenology.

[28] McDonald JC, Duffy DC, Anderson JR, Chiu DT, Wu H, Schueller OJ (2000). Fabrication of microfluidic systems in poly (dimethylsiloxane). Electrophoresis.

[29] Ghaffari Novin M, Noruzinia M, Allahveisi A, Saremi A, Fadaei Fathabadi F, Mastery Farahani R (2015). Comparison of mitochondrial-related transcriptional levels of TFAM, NRF1 and MT-CO1 genes in single human oocytes at various stages of the oocyte maturation. Iran Biomed J.

[30] Antosik P, Kempisty B, Jackowska M, Piotrowska H, Bukowska D, Wozna M (2010). Assessment of transcript and protein levels contributing to cell cycle control and gap junction connections in morphologically variable groups of porcine cumulus-oocyte complexes. Vet Med-Czech.

[31] Bukowska D, Kempisty B, Antosik P, Jaśkowski J, Olechnowicz J (2008). Selected aspects of canine oocytes maturation, fertilization and embryo development in dogs. Medycyna Weterynaryjna.

[32] Kempisty B, Antosik P, Bukowska D, Jackowska M, Lianeri M, Jaśkowski JM (2009). Assessment of zona pellucida glycoprotein and integrin transcript contents in porcine oocytes. Reprod Biol.

[33] Willadsen SM (1979). A method for culture of micromanipulated sheep embryos and its use to produce monozygotic twins. Nature.

[34] Choi W, Kim JS, Lee DH, Lee KK, Koo DB, Park JK (2008). Dielectrophoretic oocyte selection chip for in vitro fertilization. Biomed Microdevices.

[35] Cho BS, Schuster TG, Zhu X, Chang D, Smith GD, Takayama S (2003). Passively driven integrated microfluidic system for separation of motile sperm. Anal Chem.

[36] Schuster TG, Cho B, Keller LM, Takayama S, Smith GD (2003). Isolation of motile spermatozoa from semen samples using microfluidics. Reprod Biomed Online.

[37] Zeringue HC, Rutledge JJ, Beebe DJ (2005). Early mammalian embryo development depends on cumulus removal technique. Lab Chip.

[38] Hardy K, Spanos S (2002). Growth factor expression and function in the human and mouse preimplantation embryo. J Endocrinol.

[39] Díaz-Cueto L, Gerton GL (2001). The influence of growth factors on the development of preimplantation mammalian embryos. Arch Med Res.

[40] Guerin P, El Mouatassim S, Menezo Y (2001). Oxidative stress and protection against reactive oxygen species in the pre-implantation embryo and its surroundings. Hum Reprod Update.

